# Composite hemangioendothelioma of the spleen with multiple metastases: CT findings and review of the literature

**DOI:** 10.1097/MD.0000000000025846

**Published:** 2021-05-28

**Authors:** Wei wei Li, Pan Liang, Hui ping Zhao, Yan xing Zhang, Yi yang Liu, Jian bo Gao

**Affiliations:** a Department of Radiology, The First Affiliated Hospital of Zhengzhou University, Zhengzhou; b Department of Cardiology, The People's Hospital of HEBI, Hebi, Henan Province, China.

**Keywords:** composite hemangioendothelioma, hemangioendothelioma, spleen, computed tomography

## Abstract

Composite hemangioendothelioma (CHE) is a rare vascular neoplasm of intermediate malignant potential. Only 52 cases have been reported in the English literature, and one case previously reported occurred in the spleen. The purpose of our study was to report a 65-year-old man diagnosed as CHE primary arising from the spleen with multiple metastases.

Clinical and imaging features, laboratory tests, and pathological results about CHE were described in detail in this study.

The patient presented with multiple lesions in bilateral lungs and spleen that had been incidentally detected by computed tomography (CT). Except for thrombocytopenia, other laboratory tests were not significant. The CT scan of the abdomen revealed multiple round-like and irregularly mixed density masses with unclear borders in enlarged spleen. And contrast enhancement showed mild heterogeneous enhancement. CT scan also showed widespread liver, ribs, lungs, and vertebral bodies metastases. This diagnosis was confirmed by histopathological examination. The patient underwent splenectomy and still survives with tumors after six months followed-up.

Due to the lack of specificity of clinical features and laboratory tests, it is necessary to combine imaging features and pathological findings to make a correct diagnosis.

## 1 Introduction

Hemangioendothelioma (HE) is a term used to name those borderline vascular neoplasms whose clinical behaviors are between the benign hemangiomas and malignant angiosarcomas.^[1]^ HE includes various tumors with different malignant potentials.^[2]^ The members of the family of HE are intralymphatic angioendothelioma (also known as Dabska tumor), retiform HE, kaposiform HE, epithelioid HE, pseudomyogenic HE (also known as epithelioid sarcoma-like HE), and composite HE.^[1]^ Classified as an intermediate malignancy, CHE, is a rare tumor initially described by Nayler et al. in 2000.^[3]^ CHE mainly occurs in the extremities, followed by the head and neck, with the liver, spleen, and kidney are rarely reported.^[3–10]^ Most of the patients present with single or multiple purple to red papules or nodules. Treatment options include surgery, chemotherapy and radiotherapy, with total removal of the tumor is mainly implemented. Patients with CHE rarely developed metastases, but local recurrence was not uncommon. The gold standard for diagnosis is pathology, but the preoperative diagnosis is very difficult since there are no special clinical manifestations or laboratory tests. Imaging techniques like X-ray examination, computed tomography (CT), magnetic resonance imaging (MRI), and gallium scintigraphy have been used in the diagnosis of CHE.^[5,6,11,12]^ However, the literature review on imaging features has not been reported to date. In order to give radiologist a better understanding of this disease, we report a rare case of primary CHE of the spleen with multiple distant metastases and review the relevant literature.

## 2 Methods

A case of asymptomatic CHE originating from the spleen with multiple distant metastases was presented. We searched for “composite hemangioendothelioma” in PubMed and the Web of Science. A total of 110 studies that may be eligible were initially identified. 28 articles in English were included in the study with 52 patients available after exclusion of duplicate articles, abstracts, and non-relevant literature and the clinical features, imaging findings, and pathological results were summarized and discussed.

## 3 Results

### 3.1 Case report

**Figure 1:**
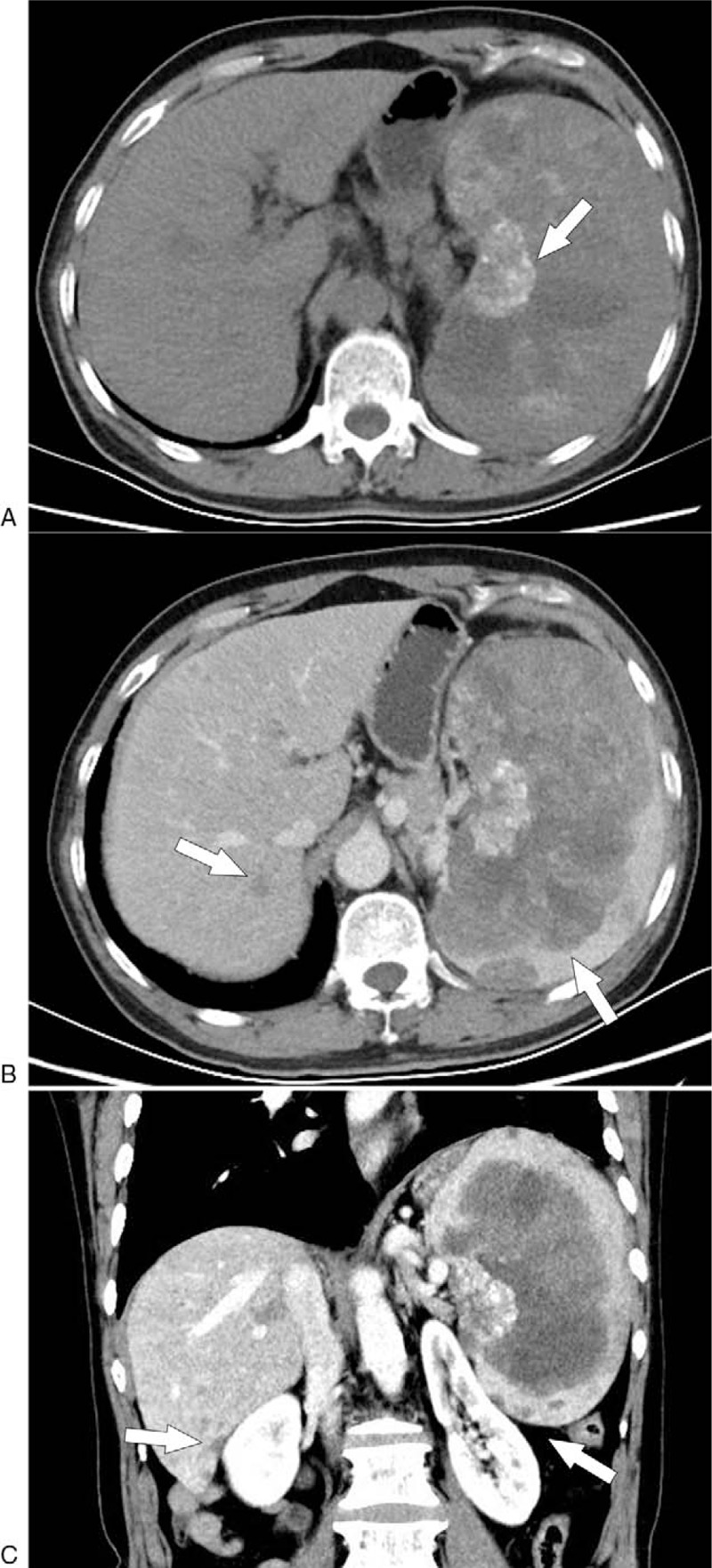
Axial: Unenhanced CT scan of the abdomen shows mixed density image with unclear boundary and cloud-like calcification in enlarged spleen (arrow) (A). Enhanced CT showed multiple slightly heterogeneous enhancement of the lesions in the spleen, and the boundary of the tumor was well-defined in the venous phase (arrows) (B). Coronal: In the venous phase, the liver is detected with multiple spotty lesions with slightly enhanced and left kidney is compressed (arrow) (C).

**Figure 2:**
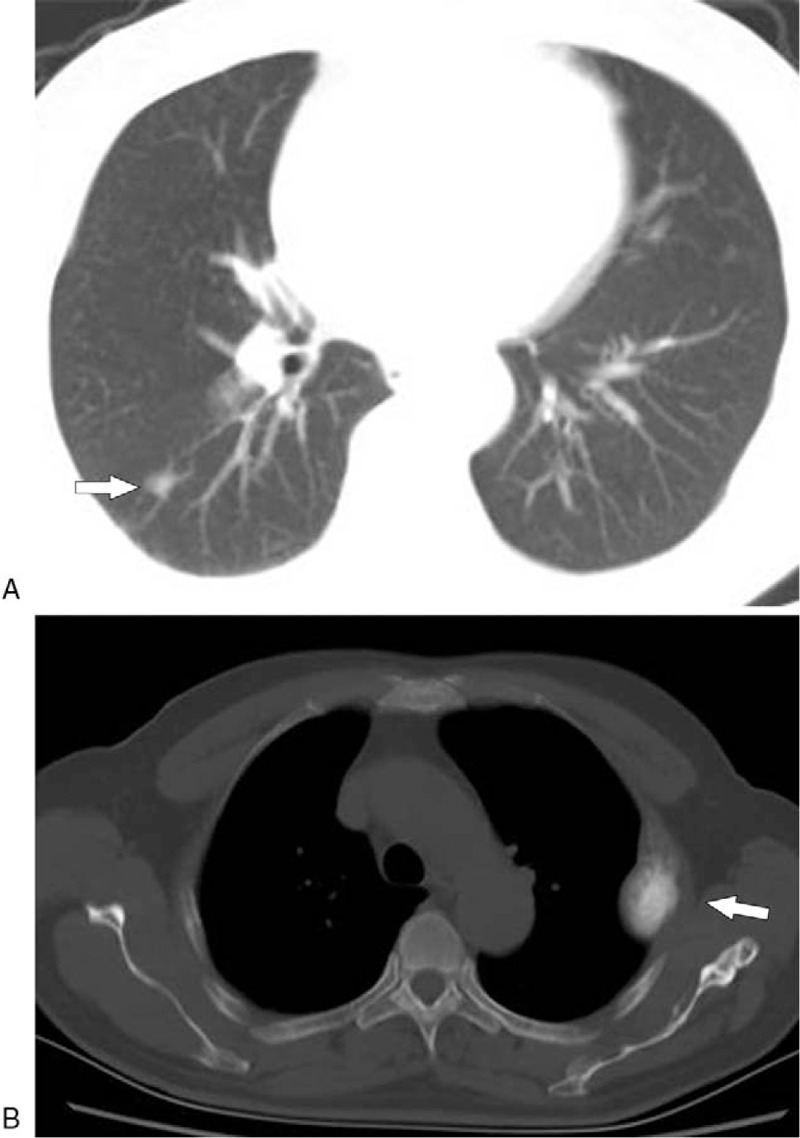
Axial: Chest CT scan shows a high-density nodule with spiculate boundary (arrow) (A). The bone window confirms the osteogenic changes of the third rib on the left side and the adjacent lung tissue being pushed (arrow) (B).

On November 4, 2019, a 65-year-old man presented at our hospital for further treatment of multiple lesions in bilateral lungs and spleen that had been incidentally detected by CT. However, the patient had no related symptoms, such as abdominal tenderness, abdominal distension, and coughing. Physical examination suggested that the spleen was 5 cm below the costal margin. The results of laboratory examination were unremarkable except for the decrease in platelet count (62 × 109/L, normal 125–350). Ultrasonography (US) showed a heterogeneous mass measuring 130 mm × 88 mm in the enlarged spleen. The boundary of the mass was poorly circumscribed, and strong echo spots could be detected. Blood flow signals were found inside the mass using Color Doppler flow imaging. Contrast-enhanced CT of the abdomen revealed multiple round-like and irregular mixed density masses in the enlarged spleen (Fig. 1B C). In addition, massive and cloud-like calcifications could also be observed in the lesions (Fig. 1A). The largest mass was measured at 152 × 85 × 132 mm^3^. In the venous phase, the boundaries were well-defined than arterial phase and pre-enhanced phase because the splenic parenchyma showed obviously homogeneous enhancement, while these masses were slightly heterogeneous enhanced or not enhanced suggesting necrosis existed. The left kidney was pushed to the other side of the spleen and deformed (Fig. 1C). Furthermore, the liver was detected with multiple spotty lesions in different sizes of low density, which were slightly enhanced after the injection of contrast materials. CT scan of the chest showed high-density nodular shadows with multiple blurred edges in both lungs (Fig. 2A). The largest of them measured 2.2 × 1.6 cm. The bone window showed local destruction of the third rib on the left, with increased density and expansive growth, pushing the adjacent lung tissue (Fig. 2B). CT scans depict the expansive growth of the fifth rib with osteoblastic changes. Also, the density of the thoracic spine, lumbar spine, and pelvis were abnormal. Multiple dot-like stone shadows could be seen at the bottom of the gallbladder. Single-photon emission computed tomography (SPECT) was subsequently performed suggesting multiple bone metastases with intense pathological radiotracer uptake in right scapula, left third rib, right fifth anterior rib, multiple vertebral bodies, sacrum, right hip joint, and left upper abdomen.

**Figure 3:**
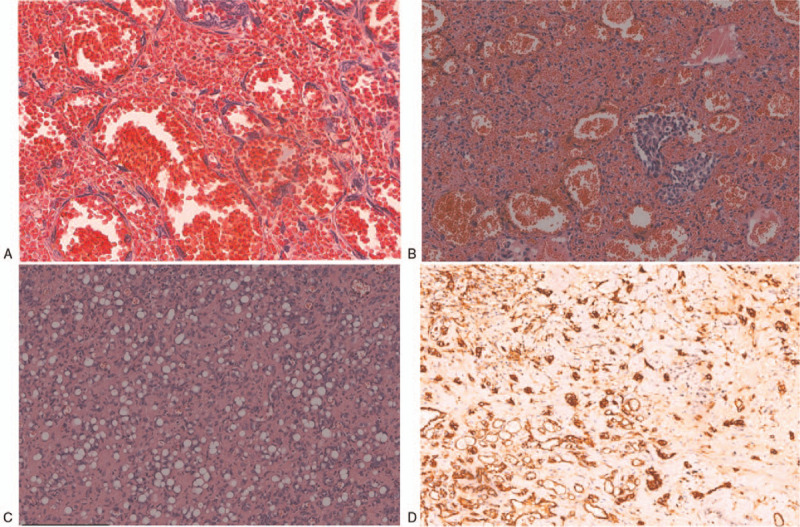
Photomicrographs showing: These irregular dilated blood vessels contain a large number of red blood cells in their lumen (H&E; ×20)(A). The proliferative endothelial cells are like papillae (H&E; ×10) (B); The tumor endothelial cells are round to oval, with large cytoplasm, eosinophilic, and often vacuolated (H&E; ×10) (C). Immunohistology showing CD31 positive endothelial cells in various areas (D) (CD31; ×10).

Fine-needle aspiration cytology of the splenic mass was performed under CT guidance, and revealed an angiogenic tumor, tending to benign or intermediate malignant lesions. Immunohistochemical results exhibited that the CD31, CD34, F-VIII, FLI-1, and ERG were positive in tumor cells. In addition, Ki67 was observed to be positive in 20% of the tumor cells. However, it showed negative results for AE1/AE3 and TFE-3. To avoid the risk of tumor rupture and thrombocytopenia, splenectomy was performed. The postoperative pathological examination confirmed the diagnosis of CHE, indicating that the grayish-white or grayish-yellow tumor in the cut surface of the resected specimen was medium-hard and that multiple grayish white or grayish red nodules with a diameter of 7 to 90 mm were observed. The histological findings displayed that the lesion was composed of hemangioma-like, epithelioid HE, and papillary intralymphatic HE (Fig. 3A and B). Based on the combination of immunophenotype and molecular detection results, the lesion was consistent with CHE.

## 4 Discussion

CHE is a very rare tumor and only 52 cases have been reported in the English literature so far. The clinical features of these patients are mentioned in Table 1. Of the 52 previously reported cases, the age of onset is from birth to 78 years, with an average age of 42.5 years. Patients with CHE showed a sex predilection of females and sites predilection of the extremities (27/52), with most patients presenting with single/multiple purple to red papules or nodules.^[3,13]^ The size of individual nodules at presentation ranged from 4 mm to 300 mm.^[6,14]^ Approximately 25% of cases occur in the head and neck, but the back,^[13,15]^ mediastinum,^[16]^ Manubrium Sterni,^[17]^ liver,^[9]^ kidneys,^[10]^ spleen,^[7]^ and other sites are rare location. Due to the low metastatic frequency and the low-grade malignant biologic behavior of the tumors patients always live with the disease for several years before the diagnosis was proposed.^[3,13]^ The clinical features and treatment of all isolated patients reported in the literature are illustrated in Table 2.^[3–30]^ The present case was a primary tumor in the spleen with multiple metastases, which was only one case reported before.^[7]^ Laboratory tests showed a slight elevation of serum CA125 level in patient previously reported with a four-month history of abdominal distension and back pain. While the patient in our case had no clinical symptoms and laboratory tests only showed a reduction in platelet count.

**Table 1:** Clinical features of CHE (N = 52) reported in the literature.

Variable	N
Gender	
Male	22
Female	30
Age, y	
Mean	42.5 ± 2.5
Range	0-78
Site	
extremities, hip	27
Head, neck	13
Back	2
Mediastinum	1
Manubrium Sterni	1
Pulmonary vein	1
Liver/Spleen/Kidney	2/1/1
Periaortic	1
Vertebral	1
Paraspinal region	1
Treatment	
Surgery	35
Surgery; chemotherapy	3
Radiation/Surgery&Radiation	2/1
Not available	11
follow-up	
NSR (3 month-7years)	24
LR/Met/LR& Met	6/4/4
Not available	14

**Table 2:** Details of clinical and treatment in patients with CHE reported in the English literature.

Author	Year	Sex/Age	Site	Size (mm)	Preoperative duration	Treatment	Outcome
Nayler et al^[3]^	2000	M/42	Foot	60	12 yr	Surgery	NSR after 1 yr
		F/27	Foot	7–20	Since childhood	Surgery	LR
		M/21	Finger	NA	Several months	Surgery	NSR after 13 yr
		M/44	Finger	10	Several years	Surgery	NSR after 2 yr
		M/70	Tongue	NA	NA	Surgery	LR, Met submandibular node and thigh
		F/31	Foot	10	2 yr	Surgery	NA
		F/71	Foot	30–40	6 yr	Surgery	NA
		M/35	Hand	30	Several years	Surgery	LR
Reis-Filho et al^[4]^	2002	F/23	Forearm, hand	130	Since infancy	Surgery	NSR after 7 yr
Sapunar et al^[5]^	2003	M/43	Toe	NA	NA	Surgery	NA
Biagioli et al^[18]^	2005	F/46	Toe	20	3 yr	Surgery	LR
Tronnier et al^[11]^	2006	F/73	Toe	31	10 yr	Surgery	LR
Fukunaga et al^[6]^	2007	F/39	Ankle, foot	300	Since birth	Partial excision	AWD
		M/44	Mandibular vestibule	13	Several months	Surgery	NSR after 13 months
		F/75	Thigh	35	10 yr	Surgery	LR
		F/37	Arm, axilla, finger, thigh	40	Since childhood	Partial excision	NA
		F/22	Foot	30	3 yr	Partial excision	NA
Fasolis et al^[12]^	2008	M/38	Oral cavity	25	NA	Surgery	NSR after 3 yr
Requena et al^[19]^	2008	M/60	Leg, Foot	NA	Since childhood	Surgery	LR, Met to inguinal lymph node
Tejera-Vaquerizo et al^[15]^	2008	F/23	Back	30	2 yr	Surgery	NSR after 30 months
Utaş et al^[20]^	2008	F/62	Forearm, hand	90	NA	Chemotherapy; surgery	NA
Aydingöz et al^[21]^	2009	F/48	Thigh	15	Several years	Surgery	LR, Met to inguinal lymph node
Cakir et al^[16]^	2009	F/50	Mediastinum	6	2 mo	Surgery	NSR after 13 mo
Cobianchi et al^[9]^	2009	F/47	Liver	90	NA	Surgery	NSR after 24 mo
Tsai et al^[22]^	2011	F/23	Foot	40	NA	Surgery	NSR after 7 mo
		F/15	Hypopharynx	32	Several months	Surgery	NSR after 18 mo
		F/49	Hypopharynx	24	Several months	Surgery	NSR after 10 mo
		M/8	Elbow	16	18 mo	Surgery	NSR after 48 mo
Chen et al^[23]^	2012	F/46	Neck	48	4 yr	Surgery	NA
Yoda et al^[7]^	2012	F/67	Spleen	NA	4 mo	Surgery; chemotherapy	Met to liver and supraclavicular lymphadenopathy
Liau et al^[24]^	2013	F/24	Scalp	15	Several months	Surgery	NSR after 1 yr
Tateishi et al^[25]^	2013	F/34	Nose	8	7 mo	Electron beam	NSR after 9 mo
Zhang et al^[10]^	2013	F/32	Kidney	26	1 wk	Surgery	NSR after 11 mo
Dong et al^[17]^	2014	M/56	Manubrium Sterni	NA	2 yr	Surgery	NA
Mahmoudizad et al^[26]^	2014	M/68	Scalp, neck	5–63	10 mo	Radiation	NA
Stojsic et al^[8]^	2014	M/58	Hip	30	Several years	Surgery	NSR after 3 mo
Leen et al^[27]^	2015	M/43	Submandibula-r area	22	3 mo	Surgery	NSR after 8 mo
Bhat et al^[13]^	2016	M/31	Back	15	1 yr	Surgery	NSR after 5 mo
Perry et al^[14]^	2017	M/47	Wrist	77	NA	NA	LR; Met to liver, lung, humerus
		F/48	Ankle	NA	NA	NA	LR
		F/36	Periaortic	21	NA	NA	Met to Sacrum
		F/48	Vertebral	NA	NA	NA	Met to Lung
		M/27	Pulmonary vein	NA	NA	NA	Met to Brain
		F/14	Ear	30	NA	NA	NA
		F/55	Hip	4	NA	NA	NSR
		M/55	Liver	69	NA	NA	NSR
		M/15	Foot	12	NA	NA	NSR
		F/59	Cheek	95	NA	NA	NA
		M/9	Finger	NA	NA	NA	NA
Rokni et al^[28]^	2017	F/78	Forehead, eye	50	18 mo	Surgery; chemotherapy	NA
Sakamoto et al^[29]^	2017	M/40	Leg, foot	20–30	6 mo	Surgery; radiation	NSR after 2.5 yr
Gok et al^[30]^	2020	M/54	Paraspinal region	26	2 yr	Surgery	NSR after 1 yr
Present case	2020	M/65	spleen	152	NA	Partial excision	ADW after 6 mo

Owing to the lack of specific clinical symptoms and laboratory tests, it is difficult to diagnose a CHE, a needle biopsy or pathological examination is required in patients. However, US, MRI, and CT can help detect masses and differentiate benign form malignant masses. US is used for screening splenic diseases because of its safety, strong discrimination against soft tissues, flexibility, and low cost. However, US is limited in identifying benign and malignant masses, describing the overall appearance of the mass, and diagnosing distant metastases. In addition, the diagnostic accuracy is closely related to the doctor's operation. Therefore, the diagnosis of CHE needs CT or/and MRI support. MRI revealed single or multiple heterogeneous lesions, which showed slightly high or high signal intensity on T2-weighted images (T2WI) and low to intermediate signal intensity on T1-weighted images (T1WI).^[9,17,22,27–30]^ After the injection of contrast medium, lesions showed moderate or strong heterogeneous enhancement on T1WI, with rare edge enhancement. Several cases have been reported that soft tissue with the lobulated surface was observed on MRI.^[22,27,28]^ Tsai et al suggested that MRI revealed unclear plantar lesions, with low dermal/subcutaneous signal intensity and moderate heterogeneity enhancement after gadolinium injection on T1WI.^[22]^ In another case reported by them, the lobulated tumor in the left pyriform sinus showed an intermediate signal intensity on T1WI, and slightly high signal intensity on T2WI, which was significantly enhanced. MRI can clearly depict muscle and soft tissue lesions, but it is not as good as CT for bone invasion and lymph node metastasis. CT revealed one or more heterogeneous and contrast-enhanced masses, with or without lobulation, lymphadenopathy, calcification.^[4,6,7,10,12,22,26,28,30]^ CT of a CHE arising from the spleen showed a large protruding cystic mass in the spleen with multiple liver nodules and supraclavicular lymphadenopathy, which was reported by Yoda et al.^[7]^ In our case, CT displayed multiple lobulated lightly enhanced or unenhanced masses of the spleen with massive calcifications. In addition, multiple metastases of the liver, ribs, lungs, and vertebral bodies were detected. These tumors were also confirmed by SPECT showing strong pathological radioactive tracer concentration in the right scapula, left third rib, right fifth anterior rib, multiple vertebrae, sacrum, right hip, and left upper abdomen. However, the liver display result was inconsistent with the CT display result, probably because the lesion was too small and the SPECT resolution was low. When CT shows bone metastasis, SPECT is recommended.

Characterized by low-grade malignancy, CHE tends to recur with infrequent metastasize, and some cases of successful treatment have been reported.^[3,4,12]^ CHE has a better prognosis than angiosarcoma. To date, only one patient has died of metastatic disease in the reported cases. Therefore, it is important to differentiate CHE from other clinically aggressive angiosarcomas preoperatively. On MRI, relative to the normal splenic parenchyma, these lesions appear as nodular hypointense on both T1WI and T2WI. Large masses with subacute hemorrhage and tumor necrosis may increase the signal intensity on both T1WI and T2WI.^[31,32]^ One or more heterogeneous complex masses of the enlarged spleen are the most common CT findings. Calcification can occasionally be seen in malignant tumors, but it may be more common in CHE. Most of the angiosarcomas present heterogeneous enhancement. Some tumors also show peripheral enhancement, with areas of decreased attenuation suggesting necrosis or bleeding.^[31–33]^ The most common metastatic site is the liver (60%), other metastatic sites include lungs, bone, bone marrow, and lymphatic system.^[32,34]^ The metastatic sites of CHE are similar to angiosarcoma. CHE that occurs in the spleen may have a potential tendency to metastasize than CHE that occurs in the superficial area because there are no clinical symptoms or the symptoms appear later. It is difficult to distinguish CHE and angiosarcoma based on image features, so it is necessary to combine clinical manifestations, laboratory examinations, and histopathological examination.

Histopathologically, CHE is characterized by a complex admixture of benign, intermediate, and malignant vascular components that occur in the deep and subcutaneous layers of the dermis, with infiltrative margins. Based on the previous studies, the most frequent histologic pattern observed in CHE is retiform HE, which is composed of long, arborizing blood vessels in a pattern resembling rete testis.^[3,28]^ Other common components including spindle cell haemangiomas (large endothelial cells with vacuolated cytoplasm with pseudolipoblastic) and epithelioid HE that typically demonstrates infiltrative chains, cords, and/or nests of epithelioid endothelial cells with lightly eosinophilic cytoplasm have also be seen in some reports. Besides, some benign components are also visible in the areas including cavernous hemangioma and arteriovenous malformations, as shown in some studies.^[10]^ Immunohistology is characterized by the positive expression of CD31, CD34, and von Willebrand factor in the tumors.^[3]^

Extensive resection treatment beyond the clinical scope is recommended for the treatment of CHE. Amputation of the affected limb can achieve better results.^[3,4]^ Other less common therapies, whether with or without resection, including radiotherapy and chemotherapy, such as electron beam, interferon-alpha 2b, and thalidomide are also effective.^[20,25,26,28]^ According to the work of Sakamoto A et al, patients with multiple tumors of the foot and sole were treated with extensive resection and radiotherapy and was followed for 2.5 years through positron emission tomography with 2-deoxy-2-[fluorine-18] fluoro-D-glucose without recurrence or metastasis.^[29]^ However, some patients do not respond to chemotherapy. Patient with primary CHE of the spleen who had undergone splenectomy and two weeks of chemotherapy did not respond to chemotherapy and were subsequently given supportive treatment.^[7]^ In our case, the patient underwent splenectomy and was provided with the best supportive care afterward. After 6 months of follow-up, the patient is still alive. Common metastatic sites include liver, lung, brain, sacrum, and lymph nodes.^[3,7,14,19,21]^ The metastasis of CHE arising from the spleen seems to be more common than that from the superficial, which may be related to the blood flow of the spleen. Compared with previous reviews, patients in our report have a worse prognosis, with local recurrence, metastasis and local recurrence &metastasis accounting for 12, 8, and 8, respectively. The reason why the metastasis rate of this article is higher may be due to the inclusion of the latest study by Perry et al. Their research includes a subset of cases that behave aggressively and that express neuroendocrine marker, which expands the scope of CHE.^[14]^ The metastasis in this article is 36%, which is much higher than that reported by Nayler et al.^[3]^ Based on the expression of neuroendocrine markers and the location of the onset, whether this subset is distinguished from “regular” CHE that behaves more inertly requires further learning.

## 5 Conclusion

The CHE of the spleen is an extremely rare vascular tumor, which is difficult to diagnose before surgery. Preoperative puncture to determine pathology is recommended when CT examination reveals splenic lobulated low-density mass with heterogeneous enhancement. Since the biological behavior is still uncertain, local excision is the first choice for treatment. In order to ensure the timely detection of recurrence and metastasis, strict follow-up is necessary.

## Author contributions

**Conceptualization:** Pan Liang.

**Supervision:** Yan Xing Zhang, Yi Yang Liu.

**Validation:** Pan Liang.

**Writing – original draft:** Wei Wei Li.

**Writing – review & editing:** Hui Ping Zhao, Jian Bo Gao.
